# Global Reach of an Internet Smoking Cessation Intervention among Spanish- and English-Speaking Smokers from 157 Countries

**DOI:** 10.3390/ijerph6030927

**Published:** 2009-02-26

**Authors:** Alinne Z. Barrera, Eliseo J. Pérez-Stable, Kevin L. Delucchi, Ricardo F. Muñoz

**Affiliations:** 1 University of California, San Francisco, San Francisco General Hospital, Department of Psychiatry, 1001 Potrero Avenue, Suite 7M, Box 0852, San Francisco, CA 94110, USA; E-Mail: alinne.barrera@ucsf.edu; 2 University of California, San Francisco, Division of General Internal Medicine, 400 Parnassus Avenue, Suite 405, San Francisco, CA 94143, USA; E-Mail: eliseops@medicine.ucsf.edu; 3 University of California, San Francisco, Helen Diller Family Comprehensive Cancer Center, Box 0128, San Francisco, CA 94143, USA; 4 University of California, San Francisco, Department of Psychiatry, Box 0984, LPPI TRC, San Francisco, CA 94143, USA; E-Mail: kdelucchi@lppi.ucsf.edu

**Keywords:** Tobacco, smoking cessation, smoking behaviors, characteristics, Web-based, Internet, international, global

## Abstract

This investigation is a secondary analysis of demographic, smoking, and depression information in a global sample of Spanish- and English-speaking smokers who participated in a series of randomized controlled smoking cessation trials conducted via the Internet. The final sample consisted of 17,579 smokers from 157 countries. Smoking profiles were similar across languages and world regions and consistent with characteristics of participants in traditional smoking cessation studies. Participants were predominantly Spanish-speakers, evenly divided between men and women and relatively few indicated using traditional smoking cessation methods (e.g., groups or medication). This study demonstrates that substantial numbers of smokers from numerous countries seek Web-based smoking cessation resources and adds to the growing support for Web-assisted tobacco interventions as an additional tool to address the need for global smoking cessation efforts.

## 1. Introduction

There are an estimated 1.3 billion smokers worldwide, of whom 650 million are expected to die prematurely of a tobacco-related disease [1]. Global mortality projections indicate that deaths attributable to tobacco use will soon be responsible for 10% of all deaths worldwide [2]. Without a doubt, tobacco use is a worldwide problem that warrants increased action focused on both tobacco control regulations and improving access to and utilization of smoking cessation interventions. Currently, there exist multiple ways to help individual smokers quit, but limited resources often impede access to these cessation aids, especially in some regions of the world.

To reach this large number of smokers across multiple countries, smoking cessation interventions are needed that can be disseminated widely and at low cost. The Internet is a viable means through which evidence-based smoking cessation interventions can be delivered [3]. The global impact of the Internet continues to grow each year, with recent reports showing that global *usage* is much higher than individual *access* [4]. Currently, countries in Asia, Europe, and the Americas report the highest access to and basic knowledge of the Internet (41.3%, 24.8%, 26.3%, respectively) [4]. However, the percent of the total population of a given region that uses the Internet is highest for countries in the Americas (73.1%) followed by countries in the regions of Oceania/Australia (57%) and Europe (47.7%). Although the rates of Internet use and access are primarily driven by certain countries within world regions (e.g., for the Americas, the USA and Canada have the greatest access), these data suggest that individuals will find a way to use the Internet even when access is not readily available.

The Internet is increasingly being recognized as a powerful tool for intervention and prevention program delivery [5]. Online interventions can reach individuals who live in remote areas where access to healthcare is limited [6] and are accessible to individuals who feel stigmatized by an illness [7]. Given the increasing number of Internet smoking cessation interventions, researchers need to examine the characteristics of individuals who decide to participate in formal outcome studies of such interventions. This will help develop evidence-based interventions for international samples of smokers using the Web to quit, as well as focus attention on those not reached, to develop campaigns that increase their participation.

A consistent pattern in the demographics of Web-based smoking cessation participants indicates that a majority of participants are White women and are highly educated. This pattern is evident in both US [8–10] and international samples [11,12]. Similar to the traditional non-Web-based samples of smokers, these smokers begin smoking between the ages of 14 to 16 years, became regular smokers at approximately 17 years, and smoke, on average, 17–20 cigarettes a day. They are interested in quitting smoking and almost all report previous quit attempts [8–12]. Thus, it appears as though smokers with a desire to quit report similar smoking characteristics, regardless of the setting where services are delivered or available.

There is an absence of reports focused on Spanish-speaking smokers, in particular, those who seek to quit via the Internet. In the USA, 15.2% of Latinos smoke [13]. Rates of tobacco use in Latin American countries are as high as 40.5% among adults and 37.9% among youths [14]. In Spain, approximately 30% of the population smokes and men between the ages of 25 and 44 years are the largest group of smokers [15]. Although there has been a global decline in tobacco use, increases in current smokers are projected to rise in developing countries. In the Americas, for example, the reported decline in the overall USA and Canadian population has been offset by projected increases in smoking expected in Spanish-speaking countries of Latin America [14]. Thus, there is a clear need to develop smoking cessation interventions that target and are accessible by Spanish-speaking smokers from all around the world.

The purpose of the current investigation is to present secondary analyses of demographic, smoking, and depression characteristics of a large global sample of English- and Spanish-speaking smokers who participated in Internet-based smoking cessation randomized trials.

## 2. Experimental Section

### 2.1. Overview

We analyzed all data available from visitors to a Website (www.dejardefumar.ucsf.edu or www.stopsmoking.ucsf.edu) conducting smoking cessation trials in Spanish and English during the period of March 1, 2002 to May 10, 2007. These automated, self-help smoking cessation Websites were designed to recruit Spanish- and English-speaking participants, obtain informed consent, collect assessment data, deliver an intervention, and conduct follow-up assessments. The sites yielded 20% quit rates at 12 months when participants with missing data at follow-up were assumed to be smoking. Complete details of the randomized trials are published elsewhere [16].

### 2.2. Recruitment

Participants for all four trials were recruited primarily through the World Wide Web. Press releases were sent to listservs, health information Websites, and written publications announcing the availability of the site and providing a link to it. Banner ads were distributed to sites that were willing to display it as a link to the study site. The studies’ URL was registered with search engines (e.g. Google, Yahoo) and Web directories using keywords such as *quit smoking* and *smoking cessation*. A Google Adwords grant provided sponsored links to the study Website and yielded a large percent of the Website’s traffic. Participants were also recruited via radio and television interviews announcing the study’s URL.

Eligibility criteria included being 18 years or older, smoking five or more cigarettes daily, using e-mail at least once per week, and planning to quit smoking within the next 30 days. Participants meeting eligibility criteria were automatically directed to the Informed Consent to Participate approved by the Institutional Review Board at the University of California, San Francisco and the University of California, San Diego. A full description of the larger randomized trials has been published elsewhere [16,17].

### 2.3. Measures

*Baseline instruments* The baseline questionnaire included demographic items, questions pertaining to smoking patterns (e.g., age smoked first cigarette and became regular smoker), the Fagerström Test for Nicotine Dependence (FTND), the Major Depressive Episode (MDE) Screener, and the Center for Epidemiological Studies Depression Scale (CES-D).

The *FTND* [18] is a six-item self-report measure that assesses level of nicotine dependence. Its range is from 0-10 with higher scores indicating a higher level of dependence on nicotine. It has shown acceptable internal consistency (*α*=0.61) and is a reliable measure of nicotine dependence and a good predictor of smoking cessation success. A mean score of 5.8 has been reported among participants in traditional randomized controlled smoking cessation studies [19].

The *MDE Screener* [20–22] is a self-report questionnaire designed to assess for the presence of MDEs based on the nine symptom criteria outlined in the *Diagnostic and Statistical Manual of Mental Disorders, 4^th^* *edition* [23]. Participants meet criteria for probable MDE if they indicate that they have experienced five or more of the nine symptoms during, at minimum, a two week period, and the symptoms interfered with their life or activities a lot; the symptoms must include either one or both of depressed mood and/or lack of interest/decreased pleasure. In a non-clinical sample of university students in Spain, the Spanish language version of the MDE Screener demonstrated good sensitivity (*κ*=0.969; 95% *CI*, 0.838–0.999), specificity (*κ*=0.967, 95% *CI*, 0.948–0.980), and accuracy (*κ*=0.968, 95% *CI*, 0.949–0.981) when compared to clinician diagnoses using the Structured Clinical Interview for DSM Disorders [22]. Additionally, the MDE Screener showed good predictive validity for quit rates in participants with past or current major depression [17,24].

The *CES-D* [25] is a self-report instrument that assesses for the presence of depressive symptoms during the previous week. Total scores range from 0–60, with higher scores indicating more severity of depressive symptoms. The national mean score in US adults [26] was 8.7 (*SD*=8.4). CES-D scores have been shown to be related to smoking status in Latinos [27] and to predict the likelihood of quitting in earlier studies [17,28].

### 2.4. Data Analyses

All analyses were conducted with the SPSS statistical software version 15.0 for Windows (SPSS Inc., Chicago, Illinois, USA). Descriptive statistics and frequency estimates were calculated for all variables. Group comparisons were based on t-test and chi-square analyses. To make this report useful to clinicians, researchers, and policy makers from diverse regions of the world, data were categorized, analyzed, and presented according to World Health Organization (WHO) regional categories (see www.who.int for countries): Africa (AFR), Americas (AMR), Eastern Mediterranean (EMR), Europe (EUR), South-East Asia (SEAR), and Western Pacific (WPR). The WHO regions are comprised of the 192 United Nations Member States. To provide additional information at the country level, data were also categorized, analyzed and presented for countries with samples greater than 500.

### 2.5. Sample

Of the 71,344 individuals screened, 48,990 participants met eligibility criteria, and 22,204 consented to participate. To be included in the analyses, participants had to have answered the item “Where do you live?” Participants who did not have data on country of residence (*n*=4515), and countries that are considered political territories (*n*=89) or are not part of the WHO regional categorization (*n*=15) were excluded from analyses. Thus, data from 17,579 (79%) participants who consented to participate in one of four trials over a five year period were included in the final analyses.

## 3. Results

### 3.1. Demographic Characteristics (Table 1)

Participants represented 157 countries. The majority were Spanish speakers (56.8%) from AMR (61.8%) and EUR (83.7%). The sample was almost evenly split between men (49.3%) and women (50.7%), although men comprised a majority of the sample in EMR (86.2%) and SEAR (95.3%). Mean age at study entry was 37 years (*SD*=10.4). A majority of participants were either married (44%) or single (30.4%) and employed full-time (63%). Participants were well educated: 52.4% had attended some or completed college, while 11.7% had obtained professional degrees. The number of participants with college and professional degrees was highest for SEAR (48.6% and 34%, respectively) and EMR (45.5% and 27.7%, respectively); these regions also reported the lowest rate of participants with less than 12 years of education. The ethnic composition of participants was predominantly White and Hispanic/Latino. For all regions except EMR and SEAR, a majority of participants identified their ethnic/racial descent as White. Hispanic/Latino participants were predominantly from AMR and EUR. All group tests were significant at *p*<0.0001.

### 3.2. Smoking Behaviors

Participants’ mean age when they smoked their first cigarette was 15.6 (*SD*=3.7) and 18.3 (*SD*=4.2) when they became regular smokers (Table 2).

The average FTND score was 5.4 (*SD*=2.4). A similar pattern of smoking behavior was reported across all the individual regions. Interestingly, however, participants in SEAR were younger (*M*=31.2, *SD*=8.2), reported an older average age when their fist cigarette was smoked (*M*=18.5, *SD*=3.6) and when they became regular smokers (*M*=20.6, *SD*=3.7), but a lower mean number of cigarettes smoked per day (*M*=13.3, *SD*=7.4) and FTND score (*M*=4.4, *SD*=2.4). All group comparisons were highly significant (*p*<0.0001).

More than half of all participants (60.7%) had not used a smoking cessation aid in the previous 6 months. Of those who did use a smoking cessation method, use of NRT aids (gum, inhaler, patch, spray) in the previous 6 months was greater for participants from WPR (24.2%) and least by participants in SEAR (8.1%). Participants in AFR endorsed the highest use of antidepressants (13.7%) to quit smoking, while a high rate of participants in EMR and SEAR indicated that they had not used any methods to quit during the previous six months (70.1% and 77.5%, respectively).

### 3.3. Depression History

Forty-seven percent reported elevated levels of depressive symptoms (CES-D score>16) during the week prior to study entry. Thirty-seven percent endorsed a history of MDE with similar reports across each of the regions. However, the distribution of current and past MDE between the samples differed such that participants from EMR reported the highest rate of current MDE (26.9%) and WPR endorsed the lowest rate (15.8%). Regarding past MDE, the rate was highest for participants in WPR (20.5%) and lowest for those residing in countries in SEAR (10.6%).

### 3.4. Smoking Behaviors and Depression History by Country

An examination of the country specific characteristics revealed similar patterns as those represented by each of the WHO regions. Of the six countries with a sample of 500 or more (see Table 3), participants from the USA included more women and were predominantly English-speaking. The remaining samples were predominantly Spanish-speaking and although the Spain sample was comprised of 53.3% women, more than 50% of the samples from Argentina, Chile, Mexico, and Venezuela were men. The USA and Mexico reported the highest rate of NRT used in the previous 6 months (23.5% and 22.5%, respectively), while Argentina reported a very low rate (4.5%). Antidepressant medications were least used in Mexico (1.4%) and most frequently used in the USA (9.8%). A notable difference was reported in the proportion of smokers who did not use any smoking cessation aids in the previous 6 months – less than 50% of the US sample. In contrast, it ranged as high as 78.7% in Chile to 62.9% in Spain.

Reports on depression history were most similar for the Spain and USA samples, in comparison to the samples from Latin America. A remarkably high and constant proportion of smokers (between 33.9% and 39.2%) screened positive for at least one MDE. However, smokers in Latin America were more likely to report a current MDE at study entry (22% to 25%) than those in the USA (16.8%) and Spain (17.3%). Consequently, the latter reported higher rates of past MDE. Mean depression symptom scores were in the elevated range for all six countries. All group comparisons were highly significant (*p*<0.0001).

Sample sizes greater than 100 and less than 500 were represented by countries in AFR, AMR, EUR, SEAR, and WPR regions; none of the EMR countries exceeded a sample size of 100. India, South Africa, and Australia represented a majority of the sample within their respective regions (92.5%, 83.2%, and 56.2%, respectively). Greater than 50% were female in samples from Canada, Uruguay, Ireland, U.K., and Australia. As expected, countries in Latin America were almost entirely comprised of Spanish-speakers, while the opposite was true of the remaining countries with samples from South Africa and India including zero Spanish-speakers. Smoking behaviors were relatively similar across countries, with the exception of reported smoking cessation aids used in the previous 6 months. The U.K., Canada, Ireland, and Australia samples were most alike in their use of methods: they reported the highest percentage of participants to use NRT aids (33.9%, 32.5%, 28.5, and 23.3%, respectively) and the lowest rate of using nothing in the six months prior to study entry (38.6%, 35.5%, 37.4%, and 38.6%, respectively); additionally, they reported higher rates of using self-help methods (11.7%, 10.0%, 15.6%, and 17.2%, respectively). The Peru sample represented the largest percentage of participants who had not used any methods to quit smoking in the previous 6 months (83.2%).

Participants from Colombia and Peru reported the highest rates of MDE history (45% and 40.8%, respectively), including current MDE (32.2% and 26.8%, respectively). Past MDE was highest in Australia (23.1%) and Canada (22.5%) and lowest in South Africa (12.9%), India (12.9%), and Colombia (12.8%).

## 4. Discussion

This investigation examined demographic, smoking and depression characteristics of a large international sample of English and Spanish-speaking smokers attempting to quit by participating in Internet-based smoking cessation randomized trials. Smokers in the current investigation were similar to community samples of smokers [8], smokers enrolled in traditional, face-to-face smoking cessation programs [29], smokers who use the Internet [10–12] and Internet users [30] in terms of gender, age, education, nicotine dependence, and number of cigarettes smoked currently. Smoking patterns were very similar across regions of the world among persons who speak English or Spanish, suggesting that smoking cessation methods might work similarly in smokers from different regions who share similar characteristics and who turn to the Internet for help in quitting. In this report, data are presented by WHO regional categorization and by country for larger sub-samples. We chose to present the data in this format to support international efforts to adapt Internet interventions to the characteristics of smokers who seek help from the Web to quit and to develop means to reach those who do not currently use these methods. We believe international health agencies currently underutilize the Web for tobacco control and other health interventions. Increasing Internet access focused on such health interventions would help scale up efforts to prevent unnecessary morbidity and mortality.

Participants from the Eastern Mediterranean and South-East Asian regions were most alike in gender composition, marital status and education. Differences between the regions can potentially be explained by the greater prevalence of male smokers [31,32] and tobacco use being viewed as a traditionally male behavior [33,34] in countries within these regions. Ten percent of women compared to 50% of men smoke in the Eastern Mediterranean [32]. Attitudes about female smoking behavior suggest greater social disapproval [35] and greater permissiveness towards alternative forms of tobacco consumption (e.g., water pipes) among family members of female tobacco users [34].

Of the 157 countries represented in this investigation, English is the national or official language of 30 countries and Spanish of 20. Other than the samples from South Africa and India, all of the countries profiled included English- and Spanish-speaking smokers, regardless of the country’s dominant language, suggesting the possibility that Internet-based cessation trials can provide a service to smokers in countries where services may not be available in their language of preference. We did not ascertain participants’ native language and the country affiliation refers to where participants reside, therefore, it is difficult to assess whether this assumption is accurate. In fact, many of the participants may be expatriates from English or Spanish-speaking countries or they may have sufficient knowledge of a non-native language to be able to use the Internet site. Nonetheless, it appears as though this smoking cessation Website reached smokers who were interested in quitting and who resided in countries where languages other than English and Spanish are the official languages.

Participants began smoking around the age of 15, became regular smokers three years later and currently smoked approximately 20 cigarettes a day. South-East Asian smokers were older when they smoked their first cigarette and when they became regular smokers, indicated fewer daily cigarettes smoked and endorsed a lower nicotine dependence score. This latter finding makes sense given their overall younger age, which translates to fewer years smoking and, possibly, less addiction. The study Website that recruited these participants is intended for individuals seeking to quit smoking cigarettes and does not address other forms of tobacco products. In India, the largest country represented in this region, *bidis*, cigarettes, and chewing tobacco are the more common forms of tobacco consumption. Approximately 65% of men and 33% of women use tobacco, but 35% and 3%, respectively, smoke [36]. The level of nicotine dependence in this investigation reflects dependence among cigarette smokers similar to that found in traditional smoking cessation studies.

Traditional smoking cessation methods were actually used by very few smokers (e.g., NRT and groups) and there were disparities in terms of utilization of smoking cessation methods between high, middle and low income countries. However, 27.5% more smokers reported a quit attempt than reported the use of smoking cessation aids in the previous 6 months, suggesting that a sizable number of participants may have used nothing (i.e., cold turkey) in their quit efforts. The countries with the largest percentage of participants using NRTs in the previous six months included the USA, Mexico, U.K., Canada, Ireland, and Australia. In these countries, NRTs are more accessible given that they are available over the counter and smokers from some of these countries are reimbursed by their country’s national health plans [14]. Except for participants from Mexico, fewer than half of participants from these countries indicated using nothing to quit, suggesting that they may have greater access to other types of cessation aids in addition to NRTs. Self-help methods were also popular among participants; however, it is unclear what types of self-help methods were used.

There is evidence that depressed individuals are more likely to begin or continue smoking and less likely to quit [37] and, consequently, tend to report higher rates of smoking behavior and nicotine dependence [38,39]. Similarly, the risk of depression onset is higher for heavy smokers than for non-smokers [40] and for current smokers versus ex-smokers [41]. Data from this investigation demonstrated that depression symptoms and clinical-level depression were very prevalent in smokers across the world. Almost 50% of the sample endorsed elevated symptoms of depression during the previous two weeks, with over a third meeting diagnostic criteria for a lifetime MDE. The rate of screening positive for current MDE reported by the entire sample was consistent with rates of current MDE among smokers in a multinational sample [41] and smokers at the start of a smoking cessation treatment program [42].

### 4.1. Limitations

There are several limitations to the data presented in this report. First, the data are representative of a specific sample of smokers who searched the Web for smoking cessation resources and were willing to participate in an online smoking cessation study and able to read in English or Spanish. Although this report included smokers from 157 countries, this report solely described characteristics of smokers who resided in countries included in the WHO regions. This study excluded smokers who reported fewer than five cigarettes per day, who are under the age of 18, who do not have Internet or email access, who are not interested in quitting, and who reside in countries or territories not included in WHO regional categorizations. Second, the sample likely had an overrepresentation of highly educated smokers who may have greater resources, including access to smoking cessation aids and interventions. Smokers with limited resources may not be aware or have access to opportunities available on the Internet to improve their health. Third, a significant portion of the English-speaking sample was from the USA, while a greater proportion of Spanish-speaking participants were from Spain. Although we obtained data on ethnic and racial background, it is difficult to draw conclusions about the cultural influences on smoking behavior and depression history given the complexity of cultural factors and its influence on behavior. Finally, this report does not include analyses on the cost-effectiveness of Web-based smoking cessation trials, which is an important factor to consider when designing such interventions.

## 5. Conclusion

This investigation demonstrates that smokers from many countries around the world are interested in participating in Web-based smoking cessation studies. Although they are from diverse cultural and linguistic backgrounds, they share similar smoking behavioral patterns. Smokers in this investigation reported characteristics that were consistent with smokers in traditional smoking cessation studies and Internet users. The results highlight the need to provide Web access to less educated smokers and to test whether these Internet interventions are also effective with populations with lower socioeconomic and educational levels. Considerable inequalities in reported use of traditional smoking cessation aids were reflected in this global sample. Web interventions that are able to achieve smoking cessation rates comparable to traditional smoking cessation methods should be championed by policy makers as an additional tool for global smoking cessation efforts.

## Figures and Tables

**Table 1:** Demographic characteristics by WHO regions, 2002-2007.

	AFR *n*=328	AMR *n*=10,673	EMR *n*=224	EUR *n*=5,478	SEAR *n*=467	WPR *n*=409
Number of countries represented / total countries comprising WHO region						
	22/46	29/35	18/21	46/52	7/11	14/27

	Percent					
Female	41.2	48.5	13.8	53.4	4.7	49.9
Language						
English	97.6	38.2	97.8	16.3	97.9	95.1
Spanish	2.4	61.8	2.2	83.7	2.1	4.9
Marital Status						
Single	36.0	29.4	39.5	36.2	50.3	27.4
Married	43.9	42.9	51.6	39.4	45.6	38.9
Separated	1.5	5.3	0.4	4.2	0.9	6.1
Divorced	5.5	9.2	3.6	3.6	1.3	9.0
Widowed	0.9	1.3	0.0	0.5	0.2	1.5
Live with partner	12.2	11.9	4.9	15.9	1.7	17.1
Education						
≤ 12 years	28.0	23.5	12.5	26.2	8.2	33.1
Some college	36.0	34.3	14.3	30.3	9.2	24.2
College graduate	28.4	29.5	45.5	29.1	48.6	32.2
Graduate degree	7.6	12.7	27.7	14.4	34.0	10.5
Employment						
Full time	75.0	60.3	72.7	69.7	74.3	60.0
Part-time	7.0	14.0	3.6	11.4	3.4	12.0
Not currently	15.0	20.7	13.4	15.8	13.7	22.3
Not ever	3.0	5.0	10.3	3.1	8.6	5.6
Income (USA dollars)						
<10,000	33.8	32.4	43.1	19.3	58.5	15.3
10,000–20,000	16.1	17.2	14.5	27.3	18.4	11.8
20,000–50,000	23.5	25.4	22.1	35.5	13.1	38.1
50,000–100,000	20.2	17.4	14.0	13.4	7.6	25.5
>100,000	6.4	7.6	6.3	4.5	2.4	9.3

Note: Data representative of participants who provided country of residence; WHO Regions: AFR – Africa; AMR – Americas; EMR – Eastern Mediterranean; EUR – Europe; SEAR – South-East Asia; WPR – Western Pacific

**Table 2:** Smoking behavior and depression characteristics by WHO region.

	AFR *n*=328	AMR *n*=10,673	EMR *n*=224	EUR *n*=5,478	SEAR *n*=467	WPR *n*=409
Smoking history *Mean* (*SD*)						
Age	34.7(10.2)	37.6(11.2)	32.4(9.0)	34.4(9.3)	31.2(8.2)	37.6(11.2)
Age, first	16.1(3.4)	15.6(3.7)	17.3(4.0)	15.3(3.1)	18.5(3.6)	15.3(3.4)
Age, regular	18.6(3.9)	18.3(4.6)	19.8(3.7)	17.8(3.5)	20.6(3.7)	17.8(4.1)
Cigarettes/day	20.8(10.7)	19.9(10.4)	20.6(10.6)	22.6(10.8)	13.3(7.4)	20.2(10.6)
FTND score	5.7(2.5)	5.2(2.5)	5.5 (2.4)	5.7(2.4)	4.4(2.4)	5.6(2.5)
Quit confidence	7.0(2.2)	7.1(2.1)	6.5 (2.4)	6.9(2.0)	6.9(2.3)	6.9(2.0)
Quit methods used in last 6 months (%)						
Methods						
NRT	13.7	16.1	14.5	16.1	8.1	24.2
Antidepressant	13.7	5.8	2.3	4.4	2.6	4.8
Alternative	3.2	3.2	2.7	3.2	0.2	6.8
Self-help	7.7	7.1	6.3	10.4	8.1	13.6
SC group	2.2	2.7	0.0	2.2	0.4	2.0
Other	4.5	3.1	4.1	3.5	3.1	3.8
None	55.0	62.0	70.1	60.2	77.5	44.7
Depression history (%)						
Major Depressive Episode (MDE)						
No MDE	69.3	62.9	60.7	63.7	64.1	63.6
Past MDE	13.2	15.8	12.3	19.1	10.6	20.5
Current MDE	17.5	21.2	26.9	17.2	25.3	15.8
CES-D (*M*, *SD*)	17.6(12.8)	17.5(12.2)	20.7(12.5)	15.8(11.9)	19.5(12.0)	17.0(12.4)

Note: WHO Regions: AFR – Africa; AMR – Americas; EMR – Eastern Mediterranean; EUR – Europe; SEAR – South-East Asia; WPR – Western Pacific; CES-D - Center for Epidemiologic Studies – Depression Scale; FTND - Fagerström Test for Nicotine Dependence; NRT – Nicotine Replacement Therapy; nicotine gum, patch, inhaler, spray; Antidepressant - Bupropion, other antidepressant; Alternative – hypnosis, acupuncture, motivational tape; SC – Smoking cessation.

**Table 3:** Smoking behavior and depression characteristics by countries with 500 or more participants.

Country	Spain *n* =4,514	USA *n* =3,852	Argentina *n* =2,005	Mexico *n* =1,605	Chile *n* =882	Venezuela *n* =531
% Female	53.3	60.4	44.7	36.2	45.0	46.1
Language						
% English	1.4	96.2	0.9	3.7	0.9	2.4
% Spanish	98.6	3.8	99.1	96.3	99.1	97.6
Smoking history *Mean* (*SD*)						
Age	34.5 (9.1)	39.3(11.6)	36.7(10.8)	36.5(11.2)	35.4(10.8)	37.8(10.4)
Age, first	15.3 (3.0)	15.5(4.4)	15.7(3.2)	15.7(3.3)	15.4(2.9)	16.2(3.6)
Age, regular	17.8 (3.5)	18.1(4.6)	18.7(4.5)	19.0(4.5)	18.9(4.4)	19.6(5.3)
Cigarettes/day	23.0(10.9)	20.3(10.0)	23.0(11.0)	17.4(9.3)	17.1(8.1)	22.1(13.2)
FTND score	5.8(2.4)	5.5(2.4)	5.4 (2.5)	4.8 (2.6)	4.7(2.5)	5.8(2.5)
Quit methods used in last 6 months (%)						
Methods						
NRT	13.8	23.5	4.5	22.5	7.6	13.5
Antidepressant	4.6	9.8	4.7	1.4	3.0	5.3
Alternative	2.9	4.4	3.3	1.3	1.7	3.4
Self-help	10.1	10.0	5.2	4.7	4.6	8.0
SC group	2.1	3.7	3.6	1.2	1.0	0.8
Other	3.5	3.7	3.1	1.9	3.4	3.1
None	62.9	44.9	74.6	67.0	78.7	65.8
Depression history (%)						
Major Depressive Episode (MDE)						
No MDE	63.4	63.7	61.2	66.1	60.8	63.3
Past MDE	19.4	19.6	14.6	11.4	15.9	11.7
Current MDE	17.3	16.8	24.3	22.5	23.3	25.0
CES-D (*M*, *SD*)	15.8(12.0)	16.2(11.8)	18.7(12.3)	17.7(12.7)	18.4(12.3)	19.0(12.6)

Note: WHO Regions: AMR – Americas; EUR – Europe; CES-D - Center for Epidemiologic Studies – Depression Scale; FTND – Fagerström Test for Nicotine Dependence; NRT – Nicotine Replacement Therapy; nicotine gum, patch, inhaler, spray; Antidepressant - Bupropion, other antidepressant; Alternative – hypnosis, acupuncture, motivational tape; SC – Smoking cessation.

## References

[1] World Health Organization Facts and figures about tobacco. First Conference of Parties to the WHO Framework Convention on Tobacco Control. http://www.who.int/tobacco/fctc/tobacco%20factsheet%20for%20COP4.pdf.

[2] Mathers CD, Loncar D (2006). Projections of global mortality and burden of disease from 2002 to 2030. PloS Med.

[3] Walters ST, Wright JA, Shegog R (2006). A review of computer and Internet-based interventions for smoking behavior. Addict. Behav.

[4] Internet World Stats Internet usage statistics - The Internet big picture world Internet users and population stats. http://www.internetworldstats.com/stats.htm.

[5] Levy JA, Strombeck R (2002). Health benefits and risks of the Internet. J. Med. Syst.

[6] Schopp LH, Demiris G, Glueckauf RL (2006). Rural backwaters or front-runners? Rural telehealth in the vanguard of psychology practice. Prof. Psychol. Res. Pr.

[7] Griffiths KM, Christensen H, Jorm AF, Evans K, Groves C (2004). Effect of Web-based depression literacy and cognitive-behavioural therapy interventions on stigmatising attitudes to depression: Randomised controlled trial. Br. J. Psychiatry.

[8] Graham AL, Bock BC, Cobb NK, Niaura R, Abrams DB (2006). Characteristics of smokers reached and recruited to an internet smoking cessation trial: A case of denominators. Nicotine Tob. Res.

[9] Lemmonds CA, Mooney M, Reich B, Hatsukami D (2004). Characteristics of cigarette smokers seeking treatment for cessation versus reduction. Addict. Behav.

[10] Stoddard JL, Auguston EM (2006). Smokers who use Internet and smokers who don't: Data from the Health Information and National Trends Survey (HINTS). Nicotine Tob. Res.

[11] Etter JF (2005). Comparing the efficacy of two Internet-based, computer-tailored smoking cessation programs: A randomized trial. J. Med. Internet Res.

[12] West R, Gilsenan A, Coste F, Zhou X, Brouard R, Nonnemaker J, Curry SJ, Sullivan SD (2006). The ATTEMPT cohort: A multi-national longitudinal study of predictors, patterns and consequences of smoking cessation; introduction and evaluation of internet recruitment and data collection methods. Addiction.

[13] Center for Disease Control and Prevention Adult cigarette smoking in the United States: Current estimates-fact sheet. http://www.cdc.gov/tobacco/data_statistics/Factsheets/adult_cig_smoking.htm.

[14] Mackay J, Ericksen M The tobacco atlas.

[15] Instituto Nacional de Estadística Encuesta Nacional de Salud de Espana 2006 [National Health Survey of Spain 2006]. http://www.msc.es/estadEstudios/estadisticas/encuestaNacional/encuesta2006.htm.

[16] Muñoz RF, Barrera AZ, Penilla C, Delucchi K, Pérez-Stable EJ Worldwide Spanish/English Internet smoking cessation trial. Nicotine Tob Res.

[17] Muñoz RF, Lenert LL, Delucchi K, Stoddard J, Pérez JE, Penilla C, Pérez-Stable EJ (2006). Toward Evidence-based Internet Interventions: A Spanish/English Web Site for International Smoking Cessation Trials. Nicotine Tob. Res.

[18] Heatherton TF, Kozlowski LT, Frecker RC, Fagerström KO (1991). The Fagerstrom Test for Nicotine Dependence: A revision of the Fagerström Tolerance Questionnaire. Br. J. Addict.

[19] Tsoh JY, Humfleet GL, Muñoz RF, Reus VI, Hartz DT, Hall SM (2000). Development of major depression after treatment for smoking cessation. Am. J. Psychiatry.

[20] Muñoz RF The Major Depressive Episode (MDE) Screener. http://www.medschool.ucsf.edu/latino/manuals.aspx.

[21] Miller WR, Muñoz RF (2005). Controlling your drinking.

[22] Vázquez FL, Muñoz RF, Blanco V, López M (2008). Validation of Muñoz’s Mood Screener in a nonclinical Spanish population. Eur. J. Psychol. Assess.

[23] American Psychiatric Association (1994). Diagnostic and Statistical Manual of Mental Disorders.

[24] Muñoz RF, Marín B, Posner SF, Pérez-Stable EJ (1997). Mood management mail intervention increases abstinence rates for Spanish-speaking Latino smokers. Am. J. Community Psychol.

[25] Radloff LS (1977). The CES-D Scale: A self-report depression scale for research in the general population. Appl. Psychol. Meas.

[26] Sayetta RB Johnson DP Basic data on depressive symptomatology: United States, 1974–1975. Vital and Health Statistics Series 11, Number 216 (DHEW Publication No PHS 80-1666); National Center for Health Statistics Hyattsville, M.D., USA 1978 10.1037/e531192008-001 7385607

[27] Pérez-Stable EJ, Marín G, Marín B, Katz MH (1990). Depressive symptoms and cigarette smoking among Latinos in San Francisco. Am. J. Public. Health.

[28] Anda RF, Williamson DF, Escobedo LG, Mast EE, Giovino GA, Remington PL (1990). Depression and the dynamics of smoking: A national perspective. JAMA.

[29] Le Faou AL, Scemama O, Ruelland A, Ménard J (2005). Characteristics of smokers seeking smoking cessation services: The CDT programme. Rev. Mal. Respir.

[30] Ono H, Zavodny M (2003). Gender and the Internet. Social Science Quarterly.

[31] Jha P, Chaloupka FJ, Carrao M, Jacob B (2006). Reducing the burden of smoking world-wide: Effectiveness of interventions and their coverage. Drug Alcohol Rev.

[32] Shafey O, Dolwick S, Guidon GE Tobacco control country profiles.

[33] Mackay J, Amos A (2003). Women and tobacco. Repirology.

[34] World Health Organization Gender and tobacco in the Eastern Mediterranean region. http://emro.who.int/ghd/PDF/gender_tobacco.pdf.

[35] Sarraf-Zagedan N, Boshtam M, Shahrokhi S, Naderi GA, Asgary S, Shahparian M, Tafazoli F (2004). Tobacco use among Iranian men, women and adolescents. Eur. J. Public Health.

[36] Chaly PE (2007). Tobacco control in India. Indian J. Dent. Res.

[37] Murphy JM, Horton NJ, Monson RR, Laird NM, Sobol AM, Leighton AH (1991). Cigarette smoking in relation to depression: Historical trends from the Stirling County Study. Am. J. Psychiatry.

[38] John U, Meyer C, Rumpf HJ, Hapke U (2004). Smoking, nicotine dependence and psychiatric comorbidity--a population-based study including smoking cessation after three years. Drug Alcohol Depend.

[39] Lee RS, Staten RR, Danner RW (2005). Smoking and depressive symptoms in a college population. J. Sch. Nurs.

[40] Klungsøyr O, Nygard JF, Sorensen T, Sandanger I (2006). Cigarette smoking and incidence of first depressive episode: An 11-year, population-based follow-up study. Am. J. Epidemiol.

[41] Wiesbeck GA, Kuhl H-C, Yaldizli Ö, Wurst FM (2008). Tobacco Smoking and Depression - Results from the WHO/ISBRA Study. Neuropsychobiology.

[42] Lugoboni F, Quaglio G, Pajusco B, Mezzelani P, Lechi A (2007). Association between depressive mood and cigarette smoking in a large Italian sample of smokers intending to quit: Implications for treatment. Intern. Emerg. Med.

